# Long non-coding RNA LINC00959 predicts colorectal cancer patient prognosis and inhibits tumor progression

**DOI:** 10.18632/oncotarget.21171

**Published:** 2017-09-21

**Authors:** Zhen-Qiang Sun, Chen Chen, Quan-Bo Zhou, Jin-Bo Liu, Shuai-Xi Yang, Zhen Li, Chun-Lin Ou, Xian-Tao Sun, Gui-Xian Wang, Jun-Min Song, Zhi-Yong Zhang, Wei-Tang Yuan

**Affiliations:** ^1^ Department of Anorectal Surgery, First Affiliated Hospital, Zhengzhou University, Zhengzhou 450052, China; ^2^ Cancer Research Institute, Central South University, Changsha 410008, China

**Keywords:** long non-coding RNA, LINC00959, colorectal cancer, epithelial-mesenchymal transition, biomarker

## Abstract

Long non-coding RNAs (lncRNAs) are increasingly implicated in tumorigenesis and cancer progression. This study focused on the relationship between the lncRNA LINC00959 and colorectal cancer (CRC). We found that LINC00959 expression was lower in CRC tissues than normal colorectal mucosae. High LINC00959 expression was negatively associated with TNM stage, distant metastasis, and lymphatic metastasis, and correlated with a better prognosis in 87 CRC cases. *In vitro*, LINC00959 knockdown enhanced colon cancer cell proliferation, invasion, and migration; upregulated N-cadherin and vimentin; and downregulated E-cadherin and Caspase-3. LINC00959 overexpression produced the opposite effects. These data suggest that LINC00959 inhibits tumor cell invasion and migration by suppressing epithelial-mesenchymal transition and promotes apoptosis through Caspase-3. LINC00959 may be a tumor suppressor and useful prognostic biomarker in CRC.

## INTRODUCTION

Global economic improvements and consequent changes in the human diet are associated with increased incidence of colorectal cancer (CRC), which is now the fifth leading cause of cancer-related death in China [1–3]. Treatment side effects, high tumor recurrence and metastasis rates, and lack of sensitive early diagnosis methods lead to poor CRC patient outcomes [4]. Multiple dysregulated genes reportedly mediate CRC tumorigenesis and progression [5], and an improved understanding of their cancer-promoting molecular mechanisms could identify novel targets for CRC oncotherapy.

Long non-coding RNAs (lncRNAs) are a heterogeneous class of non-coding RNAs >200 nucleotides in length that regulate a variety of biological interactions, such as fungal transfer RNA (ftRNA)-RNA base pairing, and RNA-protein and RNA-DNA interactions. By regulating protein-coding genes at epigenetic, transcriptional, and post-transcriptional levels, lncRNAs play important roles in tumor progression [6, 7]. Dysregulated lncRNAs were identified in diverse tumor types, including CRC [5], where they may promote or inhibit CRC development, invasion, and metastasis [8]. The present study examined lncRNA LINC00959 expression in 87 CRC cases, and assessed its prognostic value. We found that LINC00959 has potential as a novel CRC biomarker.

## RESULTS

### LINC00959 is downregulated in CRC tissues

**Figure 1:**
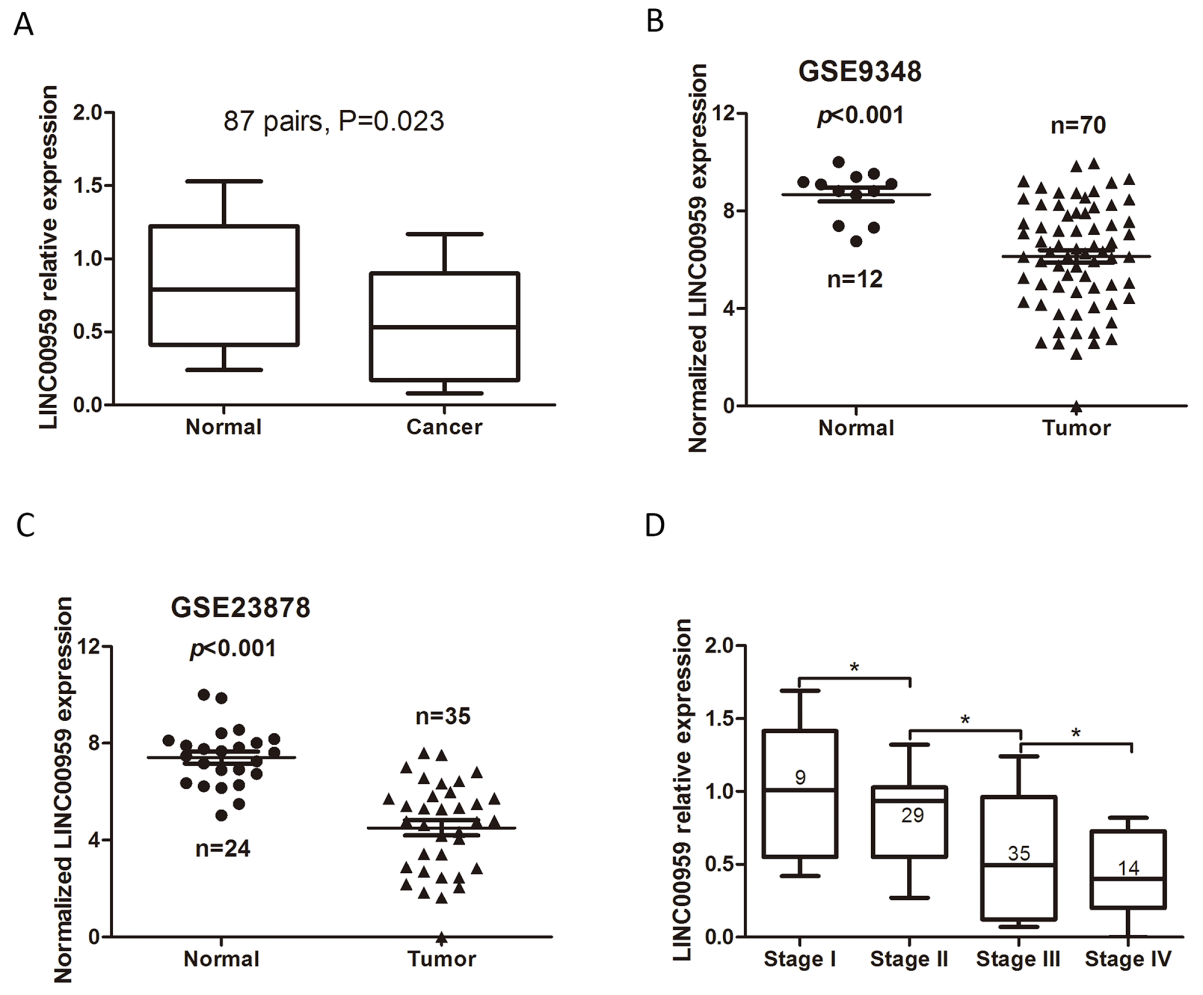
LINC00959 expression in CRC tissues qRT-PCR detection of LINC00959 in CRC tissues and normal colorectal mucosae (*P*=0.023) **(A)** LINC00959 expression in normal colorectal mucosae and CRC tissues from GEO microarray datasets (both *P*<0.001) **(B & C)** LINC00959 expression by TNM stage **(D)** *P<0.05, compared with control, using Student’s t-test.

LINC00959 was detected using qRT-PCR in tissues from 87 CRC cases, including 9 stage I, 29 stage II, 35 stage III, and 14 stage IV cases. LINC00959 expression was lower in CRC tissues than in normal colorectal mucosae (*P*=0.023, Figure 1A). We then collected LINC00959 expression information from two microarray datasets with paired CRC-normal colorectal tissues from the Gene Expression Omnibus (GEO) database. These two datasets, which used the Affymetrix HG U133 Plus 2.0 gene chip platform, were chosen because they both contained LINC00959 probes. Significance analysis of microarrays (SAM) [9] was carried out between the normal colorectal tissues and CRC samples. In the GSE9348 [10] and GSE23878 [11] of GEO datasets, LINC00959 expression was lower in CRC tissues than adjacent normal tissues (*P*<0.001, Figure 1B–1C), and high LINC00959 expression was negatively correlated with TNM stage (*P*<0.05, Figure 1D).

### LINC00959 expression is associated with the CRC malignant phenotype

The 87 CRC cases included in this study were divided into two groups, high (above mean) and low (below mean) LINC00959 expression. Relationships between LINC00959 and CRC patient clinicopathologic features were analyzed by chi-square test. High LINC00959 expression was negatively correlated with TNM stage, lymphatic metastasis, and distant metastasis (*P*<0.05, Table 1). There were no correlations between LINC00959 level and age, gender, tumor size, tumor location, or vessel invasion (*P*>0.05).

**Table 1:** Correlations between LINC00959 expression and CRC patient clinicopathologic features.

	N	LINC00959 expression		χ^2^	*P*-value
		Low	High		
**Age (years)**				0.087	0.768
≤60	48	37	11		
>60	39	29	10		
**Gender**				0.001	0.972
Male	50	38	12		
Female	37	28	9		
**Tumor size (cm)**				1.149	0.284
≤5	45	32	13		
>5	42	34	8		
**Tumor location**				0.384	0.535
Rectum	34	27	7		
Colon	53	39	14		
**TNM stage**				8.665	0.003
I/II	38	23	15		
III/IV	49	43	6		
**Lymphatic metastasis**				4.771	0.029
No	40	26	14		
Yes	47	40	7		
**Distant metastasis**				4.807	0.028
No	74	53	21		
Yes	13	13	0		
**Vessel invasion**				2.423	0.120
No	63	45	18		
Yes	24	21	3		

### High LINC00959 expression is associated with better CRC prognosis

**Figure 2:**
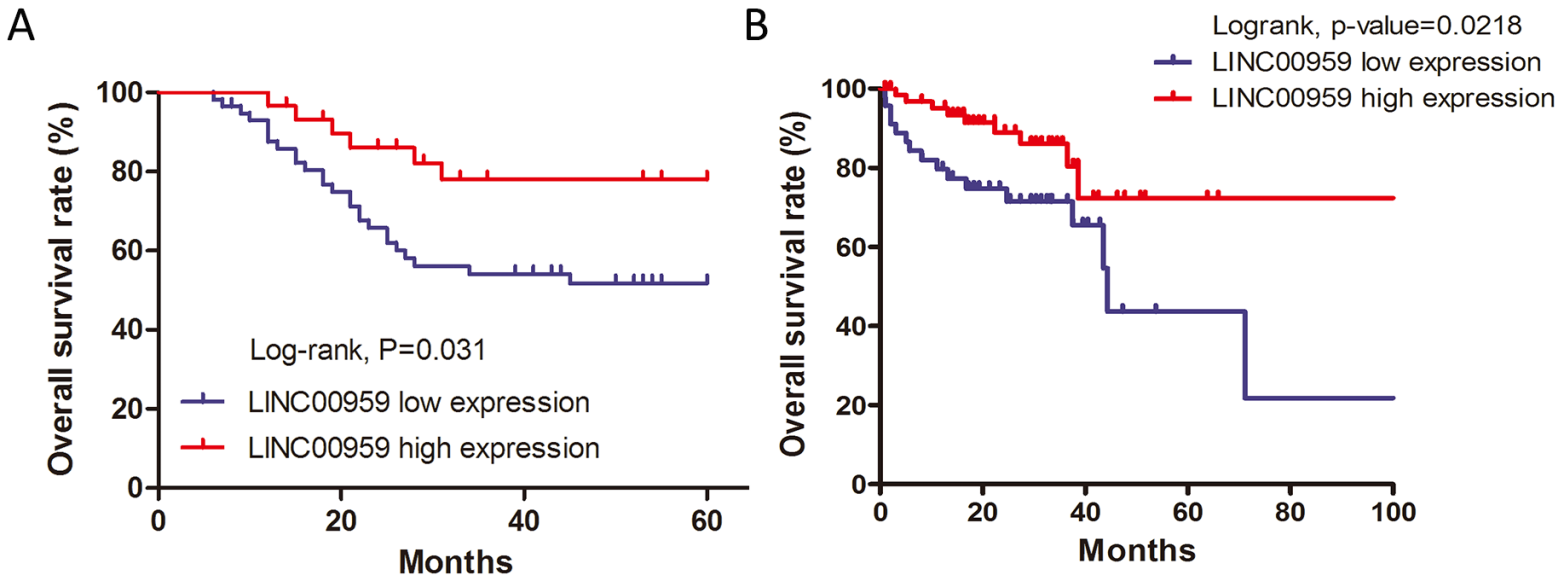
LINC00959 prognostic value in CRC patients Kaplan-Meier analysis and log-rank test were used to analyze OS in high and low LINC00959 expression CRC cases (*P<0.05) **(A)** Survival analysis for LINC00959 expression in 116 CRC patients from the OncoLnc database. (*P*=0.0218) **(B)** Correlation between 5-years overall survival rate and LINC00959 expression in 87 cases of CRC patients (*P*=0.031)

Kaplan-Meier analyses and log-rank tests in the 87 CRC cases showed that 5-year overall survival (OS) was higher in the high LINC00959 group than in the low LINC00959 group (*P*<0.05, Figure 2A). To further verify the prognostic value of LINC00959 in CRC, patient survival data and LINC00959 expression information was gathered using the OncoLnc database with TCGA survival data [12]. OS was reduced in 46 low LINC00959 expression cases compared to 70 high expression cases (*P*=0.0218, Figure 2B). According to the results of this analysis, our findings provide support for further testing of LINC00959 as a CRC prognostic indicator.

### LINC00959 expression inhibits colon cancer cell proliferation *in vitro*

**Figure 3:**
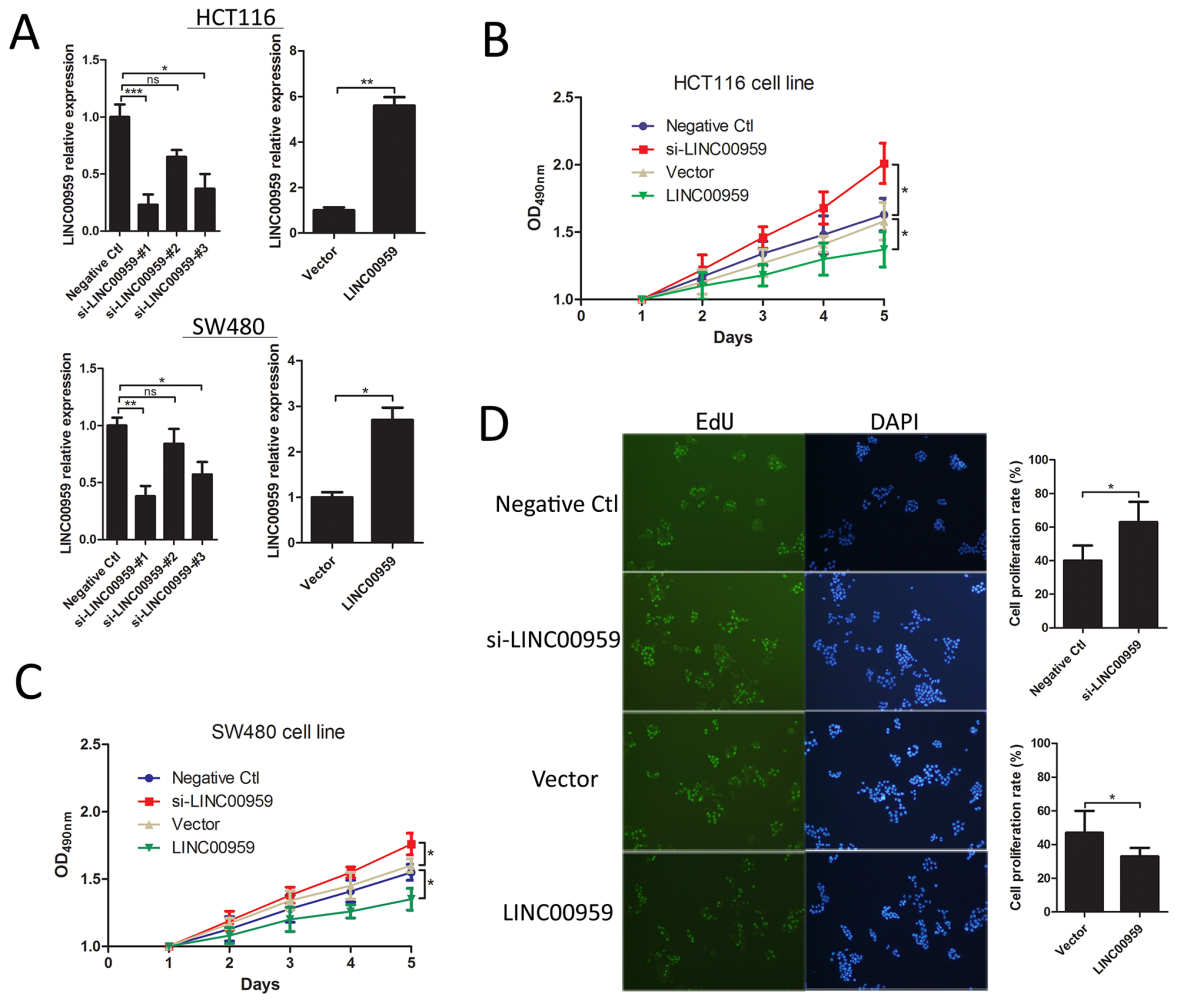
LINC00959 effects on colon cancer cell proliferation si-LINC00959, negative control, LINC00959 expression vector, or empty vector was transfected into HCT116 and SW480 cells. **(A)** Expression efficiencies and **(B** and **C)** cell proliferation as detected by MTT assay in HCT116 and SW480 cells. **(D)** EdU and DAPI staining in transfected HCT116 cells, the right panel shows numbers of proliferative and dormant cells.ns *P*>0.05, *P<0.05, **P<0.01, ***P<0.001, compared with control, using Student’s *t*-test.

HCT116 and SW480 cells were transfected with si-LINC00959, negative control siRNA, LINC00959 expression vector, or empty vector (Figure 3A). Cell proliferation was evaluated via MTT assay and EdU staining. MTT assay results showed increased proliferation in si-LINC00959-transfected cells compared to negative controls, with a significant difference after 4 d (*P*<0.05 for both cell types), and LINC00959 overexpression reduced proliferation compared to negative controls (*P*<0.05 for both cell types, Figure 3B–3C). Similarly, EdU and DAPI staining showed increased proliferation in si-LINC00959-transfected HCT116 cells compared to negative controls (*P*<0.05, Figure 3D). Our results suggest that LINC00959 inhibited colon cancer cell proliferation.

### LINC00959 expression inhibits colon cancer cell invasion and migration *in vitro*

**Figure 4:**
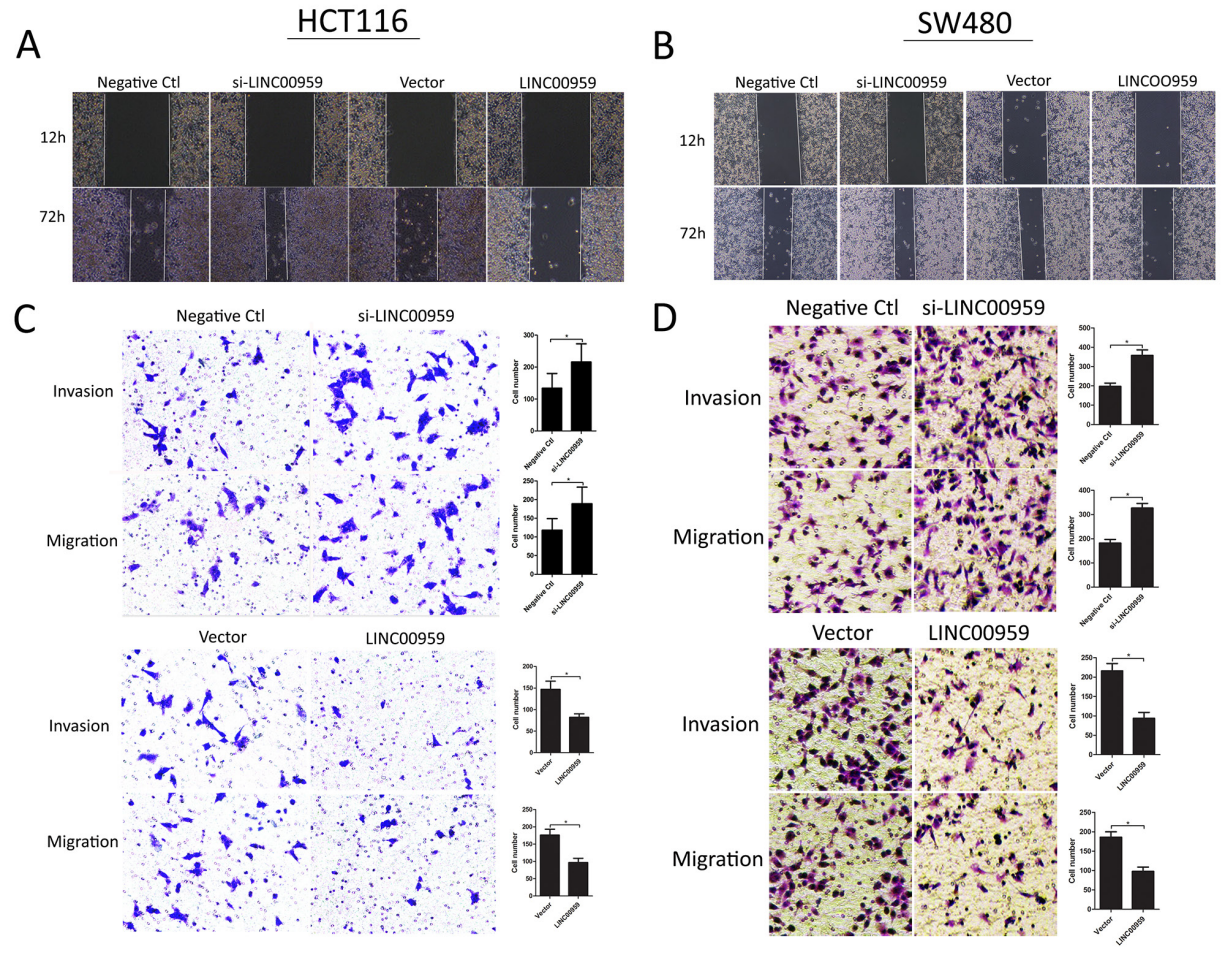
LINC00959 effects on colon cancer cell invasion and migration si-LINC00959, negative control, LINC00959 expression vector, or empty vector was transfected into HCT116 and SW480 cells. Wound-healing assays evaluated cell migration **(A** and **B)** and transwell assays evaluated cell migration and invasion **(C** and **D)** in si-LINC00959 *vs* negative control groups, and LINC00959 overexpression *vs* empty vector groups. Numbers of invading and migrating cells are shown in the right panels. *P<0.05, compared with control, using Student’s *t*-test.

Wound-healing and transwell assays were employed to assess the effects of si-LINC00959 on cell migration and invasion. Wound-healing assay results showed that LINC00959 knockdown enhanced HCT116 and SW480 cell migration, and LINC00959 overexpression inhibited migration compared to negative controls (Figure 4A–4B). Transwell assays showed that LINC00959 knockdown promoted cell invasion and migration, and LINC00959 overexpression inhibited invasion and migration (all *P*<0.05, Figure 4C–4D).

**Figure 5:**
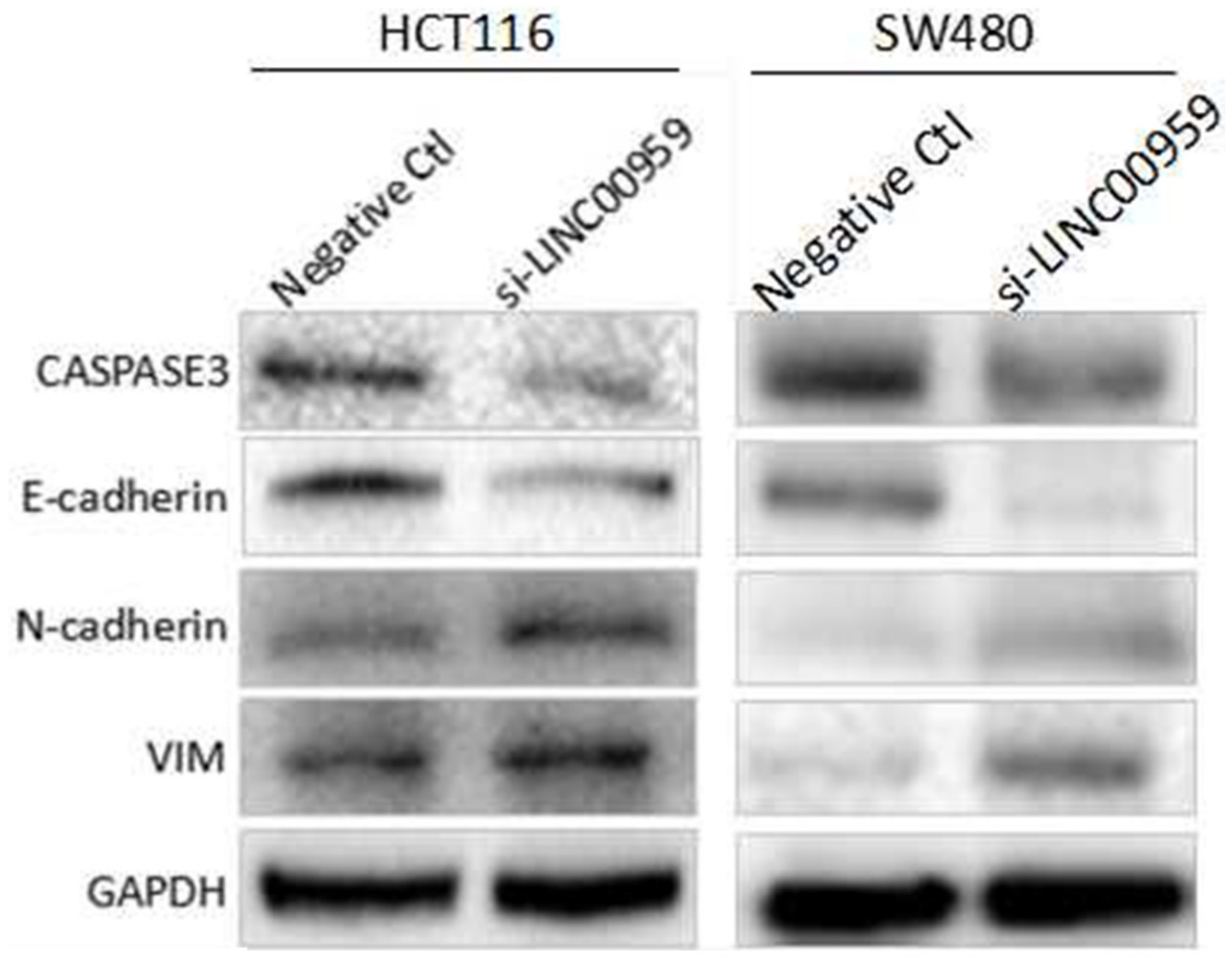
LINC00959 knockdown regulates EMT- and apoptosis-related proteins in CRC cells HCT116 cells and SW480 cells were transfected with si-LINC00959 or negative control and cultured for 48 h. E-cadherin, N-cadherin, vimentin, and Caspase-3 levels were measured by western blotting. Bands represent a typical result from three independent experiments.

The epithelial-mesenchymal transition (EMT) process is important for tumor cell invasion and metastasis [13]. We detected EMT-related molecule levels using western blotting. LINC00959 knockdown downregulated E-cadherin and upregulated N-cadherin and vimentin compared to negative controls in HCT116 cells and SW480 cells. Additionally, CASPASE-3, which mediates apoptosis, was downregulated in si-LINC00959-transfected cells compared to negative controls (Figure 5). These results suggest that LINC00959 knockdown promoted cell invasion and migration possibly by regulating EMT and/or inhibiting apoptosis *in vitro*.

## DISCUSSION

Colorectal cancer is a common malignant tumor worldwide [14]. Tumor metastasis is the leading cause of death in cancer patients [15, 16], and identifying metastatic biomarkers in tumor cells could lead to new therapeutic targets, improved diagnostic strategies. Dysregulated lncRNAs have been identified in a wide variety of human tumors using RNA sequencing [17, 18], and are associated with tumorigenesis, tumor progression, and survival in CRC patients [19, 20]. The lncRNA, LINC00959, was reportedly downregulated non-small-cell lung carcinomas, but was not previously associated with CRC [21]. In the present study, CRC patients with high LINC00959 levels had better prognoses than those with low levels, suggesting that LINC00959 may be a useful biomarker for CRC diagnosis [22].

Multiple lncRNAs have been identified as CRC oncogenes or tumor suppressors [23, 24, 25]. FEZF1-AS1 downregulation inhibits cell proliferation, migration, and invasion, suggesting that this lncRNA promotes CRC tumorigenesis and progression [26]. Qian, *et al.* discovered that NNT-AS1 is an oncogene gene in CRC; NNT-AS1 knockdown suppressed CRC cell proliferation, migration, and invasion *in vitro*, and tumor growth and metastasis *in vivo* [27]. Lnc-GNAT1-1 may suppress tumor progression, and is downregulated in CRC [28]. Our results showed that high LINC00959 expression inhibited colon cancer cell proliferation, invasion, and migration *in vitro*, suggesting that LINC00959 might be a tumor suppressor in CRC.

EMT, the process by which epithelial cells transform into more motile mesenchymal cells, is important for normal physiological processes, such as wound healing and embryogenesis, but can also promote tumor metastasis [29]. Dysregulation of EMT markers, such as E-cadherin downregulation and N-cadherin and vimentin upregulation, promotes cancer cell invasion and migration [30]. Increasing evidence suggests that lncRNAs play important roles in CRC cell invasion and migration by regulating EMT markers [31, 32]. SPRY4-IT1 knockdown inhibited CRC metastasis, possibly by upregulating E-cadherin and downregulating N-cadherin and vimentin [32]. Similarly, our study found that LINC00959 knockdown decreased E-cadherin expression and increased that of N-cadherin and vimentin. LINC00959 knockdown also decreased pro-apoptotic Caspase-3 levels [33]. Together, our results indicate that LINC00959 inhibited colon cancer cell invasion and migration, possibly by regulating EMT and promoting apoptosis through Caspase-3.

In conclusion, we identified a novel link between LINC00959 and CRC, and showed that high LINC00959 expression predicted better prognosis in CRC patients. LINC00959 knockdown promoted colon cancer cell proliferation, invasion, and migration, and its overexpression produced the opposite effects. While additional work is needed to confirm our findings, our results suggest that LINC00959 reduced cell invasion and migration by regulating EMT markers. LINC00959 may be a tumor suppressor and useful prognostic biomarker in CRC.

## MATERIALS AND METHODS

### Patient tissue samples

CRC tumor samples were collected from 87 patients during surgery at the First Affiliated Hospital of Zhengzhou University from January 2010 to June 2011. Specimens were stored at -80 °C prior to analysis. We analyzed associations between LINC00959 expression and clinicopathologic features, including patient age, gender, tumor size, tumor location, TNM stage, lymphatic invasion and migration, distant invasion and migration, and vessel invasion. Tumors were staged according to the 2010 AJCC Cancer Staging Manual [34]. Nine cases were classified as stage I, 29 as stage II, 35 as stage III, and 14 as stage IV. Study methods were approved by the Ethics Review Committees of the First Affiliated Hospital, Zhengzhou University. All patients provided signed, informed consent.

### Cell culture

HCT116 and SW480 human colon cancer cell lines were obtained from the American Type Culture Collection (USA) and cultured in DMEM and RPMI-1640, respectively, supplemented with 10% FBS (Gibco, Carlsbad, CA, USA). Cells were incubated at 37°C with 5% CO_2_.

### RNA isolation and qRT-PCR analyses

Total RNA was extracted using TRIzol Reagent (Invitrogen, Carlsbad, CA) and reverse transcription was performed using the PrimeScript RT reagent Kit (Promega, Madison, WI, USA). cDNA was amplified using SYBR Premix EX Taq™ (Takala, Dalian, China). Primers were as follows: LINC00959 forward, 5’-TGCTCCCATCCCTGCCATGT-3’ and reverse, 5’-AAGACAGGAATCTCGGGTGGGC-3’; GAPDH forward, 5’-AGCCACATCGCTCAGACAC-3’ and reverse, 5’-GCCCAATACGACCAAATCC-3’.

### Cell transfection

Small interfering RNAs (siRNAs) and a negative control were obtained from RiBo Biological Co. LTD (RiBo, Guangzhou, China). The LINC00959 overexpression plasmid and empty vector were constructed by Shanghai Jikai Chemical Technology Co. LTD (Jikai, Shanghai, China). The highest-efficiency synthesized siRNAs were as follows: siLINC00959, 5’-GGAAGGATTGATCCTAATA-3’; and negative control siRNA, 5’-GGATTAGCTAGATCGAATA-3’. siRNAs were transfected into HCT116 or SW480 cells using the Lipofectamine RNAiMAX reagent (Invitrogen, Carlsbad, CA, USA) and Opti-MEM (Gibco, Carlsbad, CA, USA) following the manufacturer’s instructions.

### Cell proliferation assays

After 48 h transfection with si-LINC00959, negative control siRNA, LINC00959 expression vector, or empty vector, 3,000 cells/well were seeded in 96-well plates for 6 h (n=5). Cell proliferation was detected via MTT assay (Roche, Basel, Switzerland) and fluorescent staining (Invitrogen, Carlsbad, CA, USA).

Transfected cells were cultured for 1, 2, 3, 4, or 5 d, and 10 μL MTT reagent was added to each well for 4 h at 37°C. Medium was then removed and DMSO was added to wells for 10 min. OD values were detected at 490 nm using a microplate reader.

For EdU staining, ClickREdU solution (Invitrogen, Carlsbad, CA, USA) was added to culture medium at a ratio of 1000:1 for 2 h to label proliferating cells. Transfected cells were washed three times with PBS, stained with 0.5 g/mL DAPI (Invitrogen, Ontario, Canada), then washed again three times with PBS. Cells were analyzed using flow cytometry (FACSCalibur DXP, BD Biosciences).

### Invasion and migration assays

Cell migration was measured using a wound-healing assay. Scrapes were created in cell monolayers in 6-well plates using a 200-μL pipette tip. Cells were washed with PBS and serum-free medium was added to wells. Wounds were photographed at 12 and 72 h to directly assess cell migration.

Transwell chambers (Sigma-Aldrich Co. LLC., St. Louis, USA) were prepared with or without matrigel, and blood serum medium with 10% FBS was added to lower chambers. Transfected HCT116 or SW480 cells were added to upper chambers for 24 h, and then residual cells in upper chambers were gently wiped away using a cotton ball. Cells were immobilized with 4% paraformaldehyde and stained with 1% crystal violet for 30 min. After three washes with PBS, cells were imaged and counted using an IX71 inverted microscope (Olympus, Tokyo, Japan). Each experiment was performed three times.

### Western blot analysis

Transfected cells were lysed using RIPA (Solarbio, Shanghai, China) to collect total protein, which was quantified via the BCA method. Protein extracts were separated on a 10% SDS-PAGE gel and transferred to PVDF membranes (GE Healthcare, Piscataway, NJ, USA). Membranes were incubated with the following primary antibodies: anti-CASPASE-3, anti-E-cadherin, anti-N-cadherin, anti-vimentin (Santa Cruz Bio-technology, Santa Cruz, CA, USA), and anti-GAPDH antibody (Cell Signaling Technology). Membranes were then incubated with second antibody and developed using an ECL kit (ECL Amersham).

### Statistical analyses

All statistical analyses were performed using SPSS version 18.0 (SPSS, Chicago, IL, USA). All experiments were performed in triplicate. Differences between LINC00959 levels were analyzed using two-sided Student’s t-test. CRC patient clinicopathological features were evaluated using the Chi-square test. Kaplan-Meier analysis and Log-rank tests were used for prognostic analyses. *P*<0.05 indicated a significant difference.
